# Microglial Contact Prevents Excess Depolarization and Rescues Neurons from Excitotoxicity^1^,^2^,^3^

**DOI:** 10.1523/ENEURO.0004-16.2016

**Published:** 2016-06-21

**Authors:** Go Kato, Hiroyuki Inada, Hiroaki Wake, Ryohei Akiyoshi, Akiko Miyamoto, Kei Eto, Tatsuya Ishikawa, Andrew J. Moorhouse, Andrew M. Strassman, Junichi Nabekura

**Affiliations:** 1 Divison of Homeostatic Development, National Institute for Physiological Sciences, Okazaki 444-8585, Japan; 2 Department of Physiology, School of Medical Sciences, University of New South Wales, Sydney 2052, New South Wales, Australia; 3 Department of Physiological Sciences, The Graduate University for Advanced Studies, Okazaki 444-8585, Nishigo-naka, Myodaiji-cho, Japan; 4 Department of Anesthesia, Critical Care and Pain Medicine, Beth Israel Deaconess Medical Center, Harvard Medical School, Boston, Massachusetts 02215; 5 Core Research for Evolutional Science and Technology (CREST), Japan Agency for Medical Research and Development (AMED), Tokyo, Japan; 6 Precursory Research for Embryonic Science and Technology, Japan Science and Technology Agency, Kawaguchi 332-0012, Saitama, Japan

**Keywords:** microglia, excitotoxicty, axonal swelling, ATP release, neuronal rescue

## Abstract

Microglia survey and directly contact neurons in both healthy and damaged brain, but the mechanisms and functional consequences of these contacts are not yet fully elucidated. Combining two-photon imaging and patch clamping, we have developed an acute experimental model for studying the role of microglia in CNS excitotoxicity induced by neuronal hyperactivity. Our model allows us to simultaneously examine the effects of repetitive supramaximal stimulation on axonal morphology, neuronal membrane potential, and microglial migration, using cortical brain slices from Iba-1 eGFP mice. We demonstrate that microglia exert an acute and highly localized neuroprotective action under conditions of neuronal hyperactivity. Evoking repetitive action potentials in individual layer 2/3 pyramidal neurons elicited swelling of axons, but not dendrites, which was accompanied by a large, sustained depolarization of soma membrane potential. Microglial processes migrated to these swollen axons in a mechanism involving both ATP and glutamate release via volume-activated anion channels. This migration was followed by intensive microglial wrapping of affected axons and, in some cases, the removal of axonal debris that induced a rapid soma membrane repolarization back to resting potentials. When the microglial migration was pharmacologically blocked, the activity-induced depolarization continued until cell death ensued, demonstrating that the microglia–axon contact served to prevent pathological depolarization of the soma and maintain neuronal viability. This is a novel aspect of microglia surveillance: detecting, wrapping, and rescuing neuronal soma from damage due to excessive activity.

## Significance Statement

Microglia, as immune cells in CNS, are highly motile cells, continuously expanding and retracting their processes as they monitor brain parenchyma. They can exert neuroprotective or neurotoxic effects, depending on their activation state. In this article, we demonstrate that microglia are attracted to overactive axons, directly connecting to these axons to reduce membrane potential and exert neuroprotection. The attraction of microglia processes depends on ATP release through volume-activated anion channels (VAACs). Blocking VAACs inhibited microglial attraction to axons, and impaired the restoration of membrane potential and axonal survival.

## Introduction

Microglia are the immune cells of the CNS, responding to disruptions of brain integrity by changing to a morphologically and biochemically distinct activated state, which then plays an important role in the inflammation and clearance of neuronal debris after cell death (Hanisch and Kettenmann, 2007; Ransohoff and Perry, 2009). Nonactivated microglia are also emerging as important elements of neuronal homeostasis in the developing and healthy brain, actively surveying the brain parenchyma and making frequent contacts with different components of neuronal circuitry (Wake et al., 2009). During development, microglia can sculpt neuronal circuits by interactions and phagocytosis, via signaling mechanisms that may include fractalkine receptors (Paolicelli et al., 2011) and complement pathways (Stevens et al., 2007; Schafer et al., 2012). These microglia–neuron contacts are more frequent and/or extensive in more active neurons (Wake et al., 2009). In larval zebrafish, such preferential microglial contacts with active neurons have been proposed to result in a selective reduction of neuronal activity (Li et al., 2012). Hence, excessive neuronal activity, such as occurs during seizures, could be expected in the short term to attract resting ramified microglia and potentially trigger their activation or functional consequences. Epileptic rodent and human brains are characterized by chronic microglial and astrocytic activation (Devinsky et al., 2013). The proliferation and activation of microglia in rodent brain begins within 3 h following chemically induced status epilepticus, but develop more strongly over the subsequent 2 d (Avignone et al., 2008). Such microglial changes can occur in the absence of clear necrosis, and reducing this activation phenotype can reduce the subsequent loss of neurons associated with status epilepsy (Ulmann et al., 2013). Coupled with the ability of microglia-derived inflammatory mediators to potentially enhance excitability, this suggests a largely detrimental role of chronic microglial activation in sustaining seizures (Devinsky et al., 2013). However, microglia can also be protective in brain pathologies (Biber et al., 2014), and, indeed, pre-conditioned activated microglia may in fact reduce seizure threshold (Mirrione et al., 2010) while nonactivated ramified microglia can also be protective against excitotoxicity in cultured neuronal models (Vinet et al., 2012). Given that most studies correlate microglia and neuronal phenotypes during chronic or prolonged hyperexcitability or excitotoxic models, we focus here on short-term interactions between microglia and hyperactive neurons, and the possible functional consequences of such interactions. We developed a novel cortical slice experimental model in which we could simultaneously measure neuronal membrane potential (*V*_m_), axonal neuronal morphology, and microglia dynamics. We show that microglia migrate to axons swollen by excessive activity, and can wrap and pinch off these affected axonal regions, thereby protecting neurons from possible excitotoxic depolarization.

## Materials and Methods

All experimental procedures involving animals were approved by the National Institute for Physiological Sciences Animal Care and Use Committee and were in accordance with National Institutes of Health guidelines. Coronal slice preparation, the shadow patch method (Kitamura et al., 2008), and two-photon imaging were used as described previously (Wake et al., 2009). Experiments were performed in 250 μm coronal slices of S1 cortex from Iba1 eGFP mice (of both sexes; age range, 28–42 d; *n* = 111; Hirasawa et al., 2005). To avoid recording from neurons near microglia activated by the brain slicing, we used neurons usually at a depth below 70 μm from the slice surface. The morphology of the microglia at this depth was comparable to that in intact brain (Stence et al., 2001). Furthermore, only a single slice in each mouse was used because microglia in subsequent slices seemed to show a gradual activation. The microglia we analyzed under these conditions were judged “nonactive,” which is consistent with previous studies (Haynes et al., 2006; Avignone et al., 2015) in that there were more than six processes and the area of the soma was <50 μm^2^.

The Krebs’ solution used for maintenance and recordings contained the following (in mm): 117 NaCl, 3.6 KCl, 2.5 CaCl_2_, 1.2 MgCl_2_, 1.2 NaH_2_PO_4_, 25 NaHCO_3_, and 11 glucose, equilibrated with 95% O_2_ and 5% CO_2._ Drugs used were tetrodotoxin (TTX; Latoxan); 6-cyano-7-nitroquinoxaline-2,3-dione (CNQX), (*RS*)-α-methyl-4-carboxyphenylglycine disodium salt [(*RS*)-MCPG disodium salt], and (*RS*)-α-methyl-4-sulfonophenylglycine (MSPG; Tocris Bioscience); and 5-nitro-2-(3-phenylpropylamino) benzonic acid (NPPB), pyridoxal phosphate-6-azo (benzene-2,4-disulfonic acid; PPADS), suramin sodium salt, and *DL*-2-amino-5-phosphonovaleric acid (AP-V; Sigma-Aldrich). Dual-color fluorescence images were collected using a two-photon laser-scanning microscope (FV-1000 MPE, Olympus) fitted with a water-immersion 40×/0.80 numerical aperture objective lens (Olympus) that was coupled to a Ti-Sapphire laser (Mai Tai DeepSee, Spectra-Physics) tuned at a wavelength of 950 nm, which simultaneously excited both Alexa Fluor 594 and eGFP. Time-lapse imaging was performed by repeated acquisition of small fluorescence image stacks comprising 15–20 focal planes, each with 0.75 μm axial spacing, and sets of images were obtained every 3 min. The spatial limitation of resolution of the two-photon imaging was 1–2 μm in the x–y-plane, and 3–4 μm for the *z*-axis. Patch pipettes (5–7 MΩ) were filled with the following (in mm): 136 K-gluconate, 5 TEA-Cl, 0.5 CaCl_2_, 2 MgCl_2_, 5 EGTA, 5 HEPES, 5 Mg ATP, and Alexa Fluor 594 hydrazide, sodium salt (Alexa Fluor 594; 80 μm; Molecular Probes). After obtaining the whole-cell configuration, 40–50 min was allowed for the diffusion of Alexa Fluor 594 into the processes. Depolarizing current thresholds for action potentials (APs) were determined using a series of 50 ms current pulses that increased in 50 pA increments. AP trains were evoked using currents with a 3× threshold, with 50 ms pulses at 10 Hz. Membrane conductance was determined in current clamp from the slope of voltage responses to current steps from −200 to +120 pA (see Fig. 4). Measurements of axonal diameter as well as axonal fluorescence intensity (FI) were made from time-lapse images acquired at 3 min intervals, from 18 min before until 24 min after the start of the 6 min stimulus train. The relative FI values of axon and microglial processes during prestimulus and poststimulus were quantified from the averaged values over the 6 frames (18 min) and 8 frames (24 min), respectively. Axonal fluorescence intensity was strongly correlated with the square of axonal diameter (*r* = 0.824, *p* < 0.001), and so axonal FI was used as an indirect measure of changes in axonal cross-sectional area.

The regions of interest (ROIs) for measurement of fluorescence intensities were defined by tracing an axon or a dendrite and including the adjacent area within a lateral distance of 3 µm. Fluorescence intensities were defined as *F*/*F*_0_ (%) = [(*F*_1_ − *B*_1_)/(*F*_0_ − *B*_0_)] × 100, where *F*_1_ and *F*_0_ are the total ROI fluorescence at a given time and at the starting time of data acquisition (*t* = −18 min), respectively; and *B*_1_ and *B*_0_ are the corresponding background FI values. Background values were taken from the darkest region of a uniformly illuminated field.

Experimental values are expressed as the mean ± SEM. Student’s *t* test was used for comparisons. A paired *t* test was used as indicated. The difference in survival rates was estimated by Kaplan–Meier analysis.

## Results

### Volume changes in the axon induced by action potential firings and dynamic behavior of microglia in the periaxonal area

**Figure 1.**
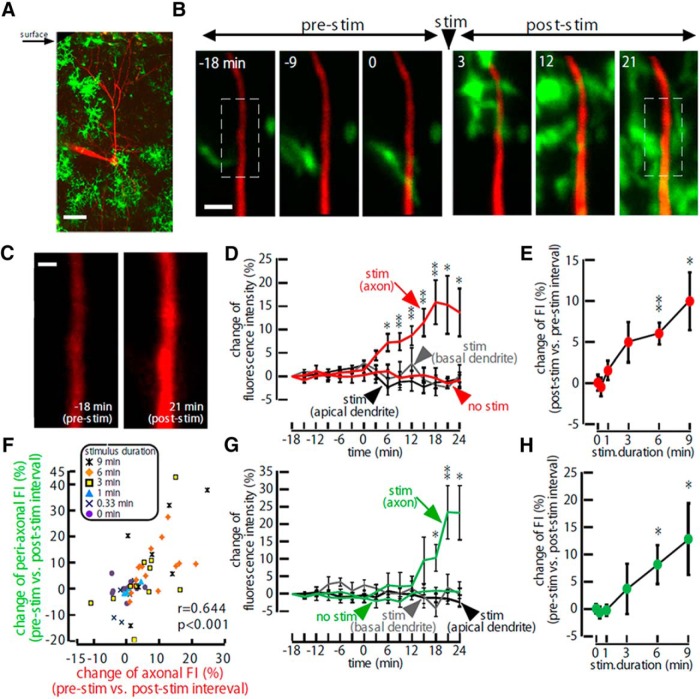
AP-induced axonal swelling and subsequent migration of microglial processes to the periaxonal area. ***A***, A low-magnification image of a cortical slice obtained from an Iba1-GFP mice in which microglia express GFP (green). Note the largely ramified morphology suggesting a “resting state,” except for some surface microglia. A recording pipette and a pyramidal neuron, from which a recording was obtained, are loaded with Alexa Fluor 594 (red). Scale bar, 50 µm. ***B***, Time-lapse images of a layer 2/3 somatosensory cortical neuron axon filled with Alexa Fluor 594 (red) and surrounding microglial processes (expressing eGFP, green), acquired at different times before and after a 3 min current stimulation protocol applied to the the soma of the neuron to evoke repetitive APs (at 10 Hz). Scale bar, 3 µm. ***C***, Higher-magnification images of axonal fluorescence signals from the areas indicated by the rectangular boxes at −12 and 6 min in ***B***. Note the increase in FI. Scale bar, 1 µm. ***D***, ***G***, Mean change in relative axonal and microglial FIs; before, during, and after 6 min of 10 Hz soma current stimulation applied from *t* = 0 (*n* = 16). Plots of the relative neuronal (red) FI (***D***) and perineuronal (green) microglial FI (***G***) for axons (including no stimulation controls) and dendritic compartments are superimposed (apical dendrite, black; basal dendrite, gray). ***E***, ***H***, Poststimulus change in relative axonal (***H***) and microglial (***H***) FIs for different durations of stimuli. ***F***, Pooled data showing the correlation between the relative stimulus-induced changes in axonal FI and microglial FI obtained from a range of 10 Hz stimulus train durations, as indicated by each symbol.

**Figure 2.**
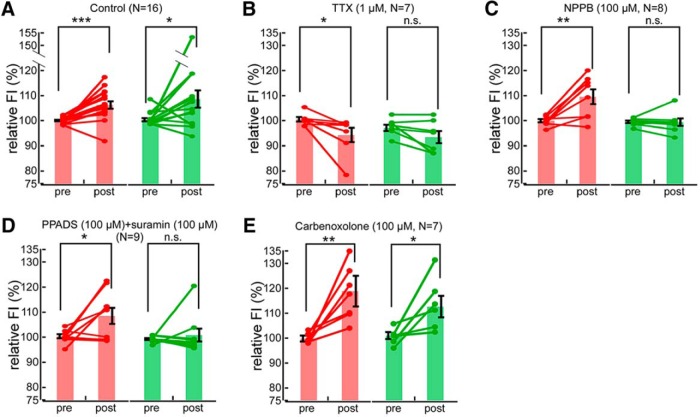
Investigating roles of VAACs in AP-induced axonal swelling and subsequent migration of microglial processes to the periaxonal area. ***A–E***, Plots of the prestimulus and poststimulus data points for axonal swelling (red) and periaxonal microglial (green) FI, for the control condition (***A***) and for each of the applied drugs (***B–E***). Individual, paired data, and mean values (±SEM) are shown. FI was averaged over the prestimulus and poststimulus time points, and these were compared using the paired *t* test: *p < 0.05, **p < 0.01, ***p < 0.001.

**Figure 3.**
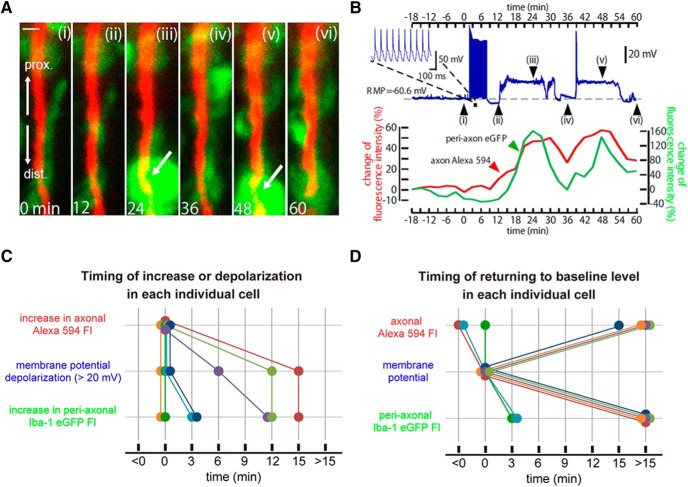
Microglial wrapping of an axon and rescuing the depolarized somatic membrane potential. ***A***, Time-lapse images of an axon (red) and surrounding microglial processes (green) before (***Ai***) and at various times as indicated (***Aii–Avi***) after a 6 min period of supramaximal AP stimulation. Scale bar, 5 µm. Arrows at ***Aiii*** and ***Av*** indicate extensive microglial accumulation around the axon. ***B***, *V*_m_ (top) and changes in relative axonal (red) and periaxonal microglia (green) FI (bottom panels) obtained from the experiment shown in ***A***. ***Bi–Bvi*** correspond to times shown in ***A***. Note that the *V*_m_ and FI are shown on the same timescale to illustrate the temporal associations among *V*_m_ depolarization, axonal swellings, and the extensive accumulation of microglial processes around the axons that precedes repolarization and reduced swelling. ***C***, ***D***, The temporal relationships among changes in axonal volume, membrane potential, and the fluorescence intensity of periaxonal microglial processes. The graphs plot data for each neuron of ***C***; the relative times of *V*_m_ depolarization and how this relates to increased axonal swelling and microglial FIs; and how the recovery of *V*_m_ relates to the recovery of axonal swelling (top) and microglia FI (bottom; ***D***). In ***C*** and ***D***, each colored line (with associated points) was obtained from a single experiment (*n* = 7). The criterion for FI increase was >5% from the prestimulus intensity, and for depolarization was >20 mV from resting *V*_m_. *V*_m_ recovery (time = 0 in ***D***) was defined as the return to within 10 mV of the initial resting *V*_m_, and microglial FI recovery (process retraction) was defined as the return to within 5% of the initial value.

To observe the dynamic behavior of microglial processes adjacent to an activated axon, we used the whole-cell patch-clamp technique to load single layer 2/3 pyramidal neurons with Alexa Fluor 594 dye in primary somatosensory (S1) cortex slices from Iba-1 eGFP mouse, and evoked trains of APs by current injection in the same neurons. Since the vast majority of microglia near the cut surface of the slice were in an activated phenotype, we used shadow patch-clamp technique (Kitamura et al., 2008) for the recording from deeper position within the slice (depth from the surface, 95.8 ± 1.2 µm; *n* = 133; Fig. 1*A*). A 6 min train of APs evoked at 10 Hz induced axonal swelling detected by an increase in FI of the red axonal Alexa Fluor 594 (increase of 5.95 ± 1.34%, *p* < 0.001; see also Materials and Methods; Figs. 1*B–E*, 2*A*). Axonal FI increases were typically accompanied by (green) FI increases resulting from recruitment of GFP-positive microglial processes (increase of 9 ± 3.42%, *p* < 0.05; Figs. 1*B*,*G*,*H*, 2*A*; Movie 1), which occurred with a delay of 0–15 min (6.25 ± 1.40 min) following the axonal FI increase (Fig. 1, compare *D*, *G*; Fig. 3*E*). No microglial soma migration was observed. Given that somatic APs can back-propagate into dendrites in cortical neurons (Stuart and Sakmann, 1994), a similar activity-induced swelling and microglial attraction may be predicted to occur in dendrites. However, the FI of apical and basal dendrites, and of the adjacent peridendritic microglial FI, did not increase (Fig. 1*D*,*G*), suggesting that microglia processes do not migrate onto active dendrites (Movie 2).

**Figure 4.**
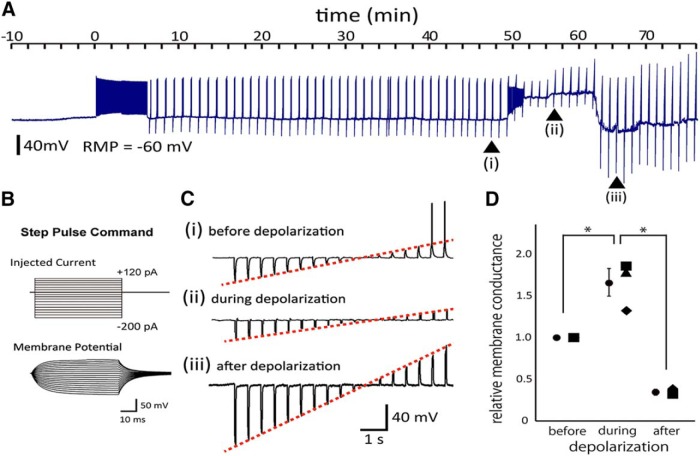
Repolarization of membrane potential is associated with a decreased membrane conductance. ***A***, Sample trace of *V*_m_ before, during, and after the application of a 6 min strong depolarizing stimulus. The regular positive and negative deflections reflect voltage responses to steps of current used to measure membrane conductance. ***Ai–Aiii*** indicate periods before, during, and after the marked spontaneous, transient depolarization. ***B***, Current pulses (top) and corresponding membrane potential responses (bottom) as used to assess passive membrane properties. Current steps ranged from −200 to +120 pA in 20 pA increments. ***C***, Representative membrane potential responses evoked by current pulses before the spontaneous depolarization (***Ci***), during the depolarization (***Cii***), and after the depolarization (***Ciii***). Red dashed lines indicate the slope of the current–passive voltage relationship used to derive the membrane conductance. Note the action potentials in ***Ci*** evoked at more depolarized potentials. ***D***, Relative membrane conductance before, during, and after the sustained depolarization derived from fitting a linear regression to the relationship between applied current and subsequent voltage responses (as shown in ***C***) to quantify the slope conductance (Δ*I*/Δ*V*_m_). Circles indicate the mean values. Individual values are shown alongside means and SEM. Means were compared using a paired *t* test. *p < 0.05

Movie 1. Time-lapse video showing an example of an excessively stimulated axon with a substantial volume increase, which was followed by intense contacts with microglial processes. Note the swollen axon being repeatedly wrapped by microglial processes, after which the substantial membrane potential depolarizations were restored to the resting levels (for details, see Fig. 4). x–y-projection images were acquired every 3 min for 78 min. 10.1523/ENEURO.0004-16.2016.video.1

Movie 2. Time-lapse video showing microglial processes migrating to an axon but not to the dendrite. The axon (bottom) was wrapped by microglial processes shortly after a substantial membrane potential depolarization was recorded. The neighboring basal dendrite (top) was not wrapped by microglia during or after this depolarization. x–y-projection images were acquired every 3 min for 60 min. 10.1523/ENEURO.0004-16.2016.video.2

Longer trains of APs (10 Hz, 0.33∼9 min) produced larger increases in both axonal and periaxonal microglial FI (Fig. 1*E*,*H*), and these increases were significantly correlated (*r* = 0.644, *p* < 0.001; Fig. 1*F*). Both increases were blocked by TTX (1 µm; Fig. 2*B*), demonstrating that they were dependent on AP generation.

### Involvement of VAACs in migration of microglial processes to swollen axons

Neuronal swelling activates volume-activated anion channels (VAACs) that can release nonexocytotic ATP (Sabirov and Okada, 2005), including from axons swollen by APs (Fields and Ni, 2010). ATP is known to be a potent attractant of microglia (Davalos et al., 2005; Haynes et al., 2006; Ohsawa et al., 2007) through the P2Y receptor; thus, we examined a potential role of VAACs and ATP release in microglial migration to swollen axons using pharmacological blockade (Fig. 2*C*,*D*). The migration of microglial processes to periaxonal regions was absent in the presence of the VAAC blocker NPPB, although AP-induced axonal swelling persisted (Fig. 2*C*). A mixture of P2-purinegic receptor blockers, suramin, and PPADS, also blocked the migration of microglial processes (premigration, 99.6 ± 0.397%; postmigration, 101 ± 2.60%; *p* = 0.560) without affecting the increase in axonal volume (Fig. 2*D*). Thus, ATP released via the activation of VAACs appears to largely mediate the attraction of microglial processes.

VAACs also function as a pathway for the release of excitatory amino acids (Okada et al., 2009), and such excitatory amino acids have been both indirectly (Li et al., 2007; Fontainhas et al., 2011) and directly (Liu et al., 2009) reported to increase microglial process motility. Carbenoxolone also failed to block the axonal volume increase and migration of microglial processes to axons (Fig. 2*E*), suggesting that the activation of gap junction hemichannels (Li et al., 2012) was not a key contributor to the release of chemoattractants induced by axonal volume increase.

### Microglial wrapping of damaged axons rapidly reverses the pathological depolarizations of neurons

In a subset of neurons (7 of 38, 18%), the stimulation-induced axonal swelling was associated with large (>20 mV) and rapid depolarizations of the soma *V*_m_ (Fig. 3*B*). The reversal of the depolarization was observed in every case following the migration and wrapping of microglial processes around the swollen axon (Fig. 3*A–D*). Some neurons had an additional cycle of depolarization and repolarization, in association with an additional episode of axonal swelling and microglial wrapping (Fig. 3*B*). To clarify the temporal relationship between the changes in axonal volume, *V*_m_ fluctuations, and microglial processes migration, we compared (1) the onset of the increases in axonal and microglial FI and pathological *V*_m_ depolarizations, and (2) the times when these three values returned to their baseline levels. Pooled data suggested that the increase in microglial FI never preceded the axonal FI increase or the depolarization (Fig. 3*C*; *n* = 7). Similarly, depolarization never preceded axonal FI increases. Complete retraction of microglial processes away from the axon, as reflected in the return of periaxonal microglial FI to baseline (change of <5%), did not occur until after the recovery of the *V*_m_ to resting values (Fig. 3*D*; *n* = 7). Together, the temporal relationships are consistent with axonal swelling triggering both *V*_m_ depolarization and microglial process accumulation, while *V*_m_ recovery occurs during the microglial contacts. From these results, we proposed that, in response to excessively swollen axons, microglia make intimate contacts or wrapping of axons to “rescue” the neuronal somas from further progressive depolarizations. This rescue could be observed in response to the accumulation of microglial processes alone (Fig. 3*A*,*B*). The repolarization induced by microglial wrapping could be due to changes in ionic current flowing across the axonal or soma membrane. In three neurons to which current steps were applied to examine the change in the membrane conductance during depolarization and after repolarization (Fig. 4*A*,*B*). The membrane conductance was significantly increased during depolarization and reduced after the sequential repolarization (Fig. 4*C*,*D*). This suggests channels or a leak mediating inward current close upon the membrane repolarization, or that the soma becomes electrically uncoupled from regions of the affected axons.

**Figure 5.**
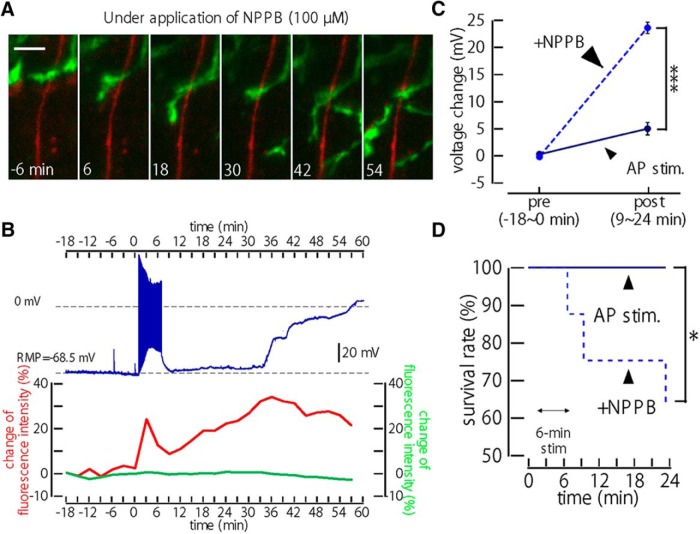
Inhibition of microglial migration to swollen axons by block of VAACs. ***A***, Time-lapse representative images of an axon (red) and microglial processes (green) at different times before and after a strong (6 min) depolarizing stimulus, all in the presence of VAAC block. Scale bar, 5 µm. ***B***, Representative traces of *V*_m_ and axonal and microglial FIs in the presence of NPPB, from a different neuron as shown in ***A***. A sudden, large depolarization followed the stimulation-induced increase in axonal FI (red), but no increase in microglial FI (green) was seen adjacent to the axon, and no recovery of the depolarized *V*_m_ was seen (*V*_m_ continued to further depolarize to 0 mV). ***C***, Pooled data showing the mean change of *V*_m_ following 6 min of AP stimulation, in control conditions (*n* = 16, solid line) and in the presence of NPPB (*n* = 8, dashed line). ***p < 0.005. ***D***, Kaplan–Meier survival curves of neurons treated with NPPB (*n* = 8, dashed line) and in control (*n* = 16). *p < 0.05. Neuronal “death” was defined as a neuron whose *V*_m_ depolarized to close to 0 mV for more than a few minutes (typically followed by apparent loss of the Giga seal).

Finally, we compared *V*_m_ depolarizations with and without NPPB, which blocks the microglial–axon interactions (Figs. 1*J*,*M*, 5*A*,*B*). The averaged data showed that, following a long 6 min 10 Hz AP stimulus, a larger mean *V*_m_ depolarization was observed in the presence of NPPB (Fig. 5*C*; *n* = 16 for control; and *n* = 8 for NPPB), which progressed to the complete loss of the *V*_m_ (∼0 mV; Fig. 5*B*) and resulted in the acceleration of neuronal death with NPPB (Fig. 5*D*; *p* < 0.05).

## Discussion

We have developed an acute experimental model for studying the role of microglia in CNS excitotoxicity that allows us to simultaneously examine excitotoxic effects on axonal morphology, neuronal membrane potential, and microglial migration induced by suprathreshold stimulation of individual S1 cortical layer 2/3 pyramidal neurons. We used this model to directly demonstrate that microglia exert a short-term and highly localized neuroprotective action under conditions of neuronal hyperactivity. The evoking of repetitive APs in individual pyramidal neurons resulted in selective swelling of axons, but not dendrites, which was accompanied in some cases by a large and sustained depolarization of somatic membrane potential. Microglial processes migrated to these swollen axons in a mechanism involving glutamate and ATP release via VAACs. This migration to swollen axons was followed by intensive microglial wrapping that induced a rapid somatic membrane repolarization back to its resting value. When the microglial migration was pharmacologically blocked, the activity-induced depolarization continued until cell death ensued, demonstrating that microglia–axon contact served to prevent pathological depolarization of the soma and maintain neuronal viability.

VAACs are ubiquitously expressed and activated by swelling, playing an important role in regulatory volume decreases in swollen cells (Okada et al., 2009). The release of ATP through axonal VAACs activated by swelling during trains of APs has been directly demonstrated in cultured dorsal root ganglion neurons (Fields and Ni, 2010). Both P2X and P2Y ATP receptors are expressed on microglia (Xiang and Burnstock, 2005), and ATP induces the migration of microglial processes toward the source of ATP (Davalos et al., 2005; Wu et al., 2007). Our result that NPPB and P2-purinergic receptor antagonists both block axonal swelling-induced microglial migration supports the idea that VAACs are activated by AP-induced swelling and release of ATP to induce microglia chemotaxis to swollen axons. Given that VAACs are also permeable to glutamate (Liu et al., 2006) and microglia express a variety of glutamate receptors (Murugan et al., 2013), glutamate may additionally contribute to microglial–axon interactions. Excitotoxicity typically involves necrotic neuronal damage or death in conjunction with disruptions in cell volume and its regulation (Okada et al., 2009). In such models, microglia can be either neurotoxic (Yrjanheikki et al., 1998;Cai et al., 2006; Biber et al., 2014) or neuroprotective (Neumann et al., 2006; Hulse et al., 2008; Biber et al., 2014). However, these studies have assessed neuronal viability, histological damage, and/or behavioral parameters at least a day after the onset of the damaging stimuli. The acute early microglial responses observed here, including the engulfment of axons, may offer protection via physical shielding of leaky axonal membranes, as proposed for leaky cerebral vessels (Nimmerjahn et al., 2005), by releasing neuroprotective signaling molecules (Kettenmann et al., 2011) and/or by phagocytosing damaged portions of axons to minimize the release of cytotoxic intra-axonal contents (Streit, 2002; Neumann et al., 2006). The decreased membrane conductance observed during the axonal repolarization indicates less permeability to ions with a more depolarized equilibrium potential, either through selective closure of channels or by electrical uncoupling from leaky axons (e.g., by sealing membrane damage). The functional consequences of this microglial response resemble a first-aid approach whereby they “bandage” or “excise” the damaged axons. The microglial first aid may also function as a “circuit breaker” that prevents overexcitation of postsynaptic neurons to the hyperactive axons. Resting microglia can physically interact with neuronal elements in an activity-dependent manner in healthy brain (Wake et al., 2009), and these interactions reduce hyperactivity of neurons (Li et al., 2012). Our results reveal another facet to the mechanisms by which microglia detect abnormal neuronal structure or function and respond to maintain neuronal circuit homeostasis.

## Synthesis

The decision was a result of the Reviewing Editor Alfonso Araque and the peer reviewers coming together and discussing their recommendations until a consensus was reached. A fact-based synthesis statement explaining their decision and outlining what is needed to prepare a revision is listed below.

I concur with the reviewers' comments expressing the novelty and quality of the study. And I also consider pertinent the concerns expressed. I believe that addressing those concerns, which is probably relatively easy and will definitely improve the manuscript, is necessarily required to properly support the conclusions.

Specific comments of the reviewers:

Reviewer 1.

In the present work, using two photon imaging and electrophysiological techniques, authors showed that action potential firing induced axonal swelling and subsequent migration of microglial processes due to VACCs, P2, glutamate receptors in cortical slices of SI cortex.

The experimental design and the results presented in the manuscript are very simple and straight forward, and most of the conclusions reached sufficiently supported by experimental data.

Results are mostly convincing and their discussion is accurate. Nevertheless, some specific issues need to be clarified:

Pag 2, L 25: The author claim. "we demonstrate microglia attract on to the firing axons and have direct connection to reduce membrane potential exerting neuro-protective effect" So, How microglia can reduce the membrane potential? What is the mechanism?

Pag 4, L 74: The authors should clarify the material and methods i.e. To reduce recording from activated mice (?) (as judged by ameboid morphology and reduced processes), we used neurons deeper within the slice. How deep the cells were recorded? This information should be given in this section

How was "non active" microglia determined?

Pag 6, L 135: Image/movie showing that microglia processes do not migrate to the dendrites is welcome.

Data reported in Figs 3-4 seems to correspond to individual experiments. But the averaged data from multiple experiments should be reported. Also, at least one control condition should be showed (i.e. without neuronal hyperactivity)

Reviewer 2.

The study has been carefully performed. The manuscript is well written, although there are a few typos; e.g. line 226: phagocytozing not phagocytizing.

The data are convincing. However, more data should be provided on some important aspects.

The authors discuss a role for axonal glutamate release, however, they do not provide respective data. Therefore, more experiments should be performed to clarify the impact of glutamate. Alternatively, the authors should down-tune the glutamate aspect in their function.

The pathway of purinergic signals from axons to microglia is supported by strong pharmacological experiments. In contrast, we are still missing equivalent experiments explaining at the molecular level how microglial ensheathment of swollen, hyperactive axons causes efficient repolarization. Experiments addressing the physiological mechanism that leads to repolarization could significantly enhance the impact of this study. E.g. are microglial cells involved in potassium regulation, do they restrict the extracellular space, or are there other microglial messengers that affect determinants of sodium/potassium regulation (Na+/K+ ATPase or K+ channels) in the axon.

Figure 3 C and D including the legends are very hard to understand and should be improved. Using different colors per experiment rather than solid black lines could be helpful.
